# The effect of reducing dietary energy on performance, intestinal morphology and intestinal peptide and amino acid transporters in broiler chicks

**DOI:** 10.1002/vms3.1364

**Published:** 2024-01-31

**Authors:** Reza Mahdavi, Shahab Ghazi Harsini, Ali Hossein Piray

**Affiliations:** ^1^ Department of Animal Science, College of Agriculture and Natural Resources Razi University Kermanshah Iran

**Keywords:** amino acid transporters, broilers, intestinal morphology, metabolizable energy, peptide transporter

## Abstract

**Background:**

Previous research has investigated the impact of different factors such as gender and age of birds, protein and amino acid levels, protein origin and the physical form of the diet, on the mRNA expression of intestinal peptides and amino acid transporters. Nevertheless, there have been no attempts to examine the impact of dietary energy density on the mRNA abundance of intestinal amino acid and peptide transporters in humans or animals. This is the first reported case in both humans and animals.

**Objectives:**

This experiment aimed to evaluate the influence of two levels of metabolizable energy ([ME]; 2950 and 2850 kcal ME/kg diet) on performance and jejunal morphology and mRNA expression of amino acid (B0AT, b^0,+^AT, CAT1 and y^+^LAT1) and peptide (PepT1) transporters in broiler chickens during the starter period (0–10 days of age).

**Methods:**

Two hundred and seventy day‐old male Ross 308 broiler chickens were randomly assigned to two dietary treatments with nine replicates and fifteen birds in each replicate. At 10 days of age, the outcome variables were measured, which included the assessment of the mRNA abundance of PepT1, b^0,+^AT, B0AT, y^+^LAT1 and CAT1 in the jejunal section using real‐time PCR.

**Results:**

The findings of this study revealed no significant impact of the dietary treatments on feed intake, body weight gain, feed conversion ratio and the jejunal mRNA abundance of PepT1, CAT1 and y^+^LAT1 (*p* > 0.05). However, a reduction in dietary ME level from 2950 to 2850 kcal ME/kg diet resulted in a decrease in the mRNA level of B0AT and b^0,+^AT (*p* ≤ 0.05). The reduction in dietary energy level did not lead to any significant changes in villus width, villus height, crypt depth, villus surface area and villus height to crypt depth ratio (*p* > 0.05).

**Conclusion:**

The results demonstrated that decreasing dietary energy density may alter the expression of amino acid transporters in the intestine while having no impact on the growth performance and morphometric structure of the intestine.

## Graphical abstract

This study was conducted to assess the effects of dietary energy level on the mRNA expression of five genes (B^0^AT, b^0,+^AT, CAT1, y^+^LAT1 and PepT1) and intestinal morphology of broilers chicks. Performance and intestinal morphology were not affected by dietary treatments. Jejunal mRNA levels of PepT1, CAT1 and y^+^LAT1 were not affected by dietary energy level, but mRNA abundance of the B^0^AT and b^0,+^AT decreased as ME level decreased from 2950 to 2850 kcal/kg.

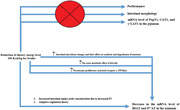

## 1 INTRODUCTION

The productivity of modern commercial broiler strains is heavily reliant on the first few days of their life (Payte et al., 2022) as they grow rapidly and attain slaughter weight within a few weeks. The productivity of broiler chickens is highly dependent on post‐hatch nutrition, given that digestive tract development and muscle cell proliferation occur during the starter period (Ravindran & Abdollahi, 2021; Li et al., 2022). The transition from the embryogenic to the post‐hatch phase is the most challenging stage in broiler breeding, as it involves significant physiological and morphological changes in the gastrointestinal tract (GIT) required for effective nutrient digestion and absorption by the chick (Vieira & Moran, 1999; Willemsen et al., 2010). The expression of genes involved in growth control undergoes significant changes simultaneously with substantial alterations in GIT development (Gilbert et al., 2010; Osmanyan et al., 2018). Factors such as bird gender and strain, dietary nutrient density, disease condition and feed form are key elements that influence GIT development (Ravindran &Abdollahi, 2021).

Energy is the most costly component of poultry diets. Carbohydrate, fats/oils and occasionally dietary protein sources are used to meet the bird's energy requirements, with fats/oils playing a crucial role as an energy source in poultry diets (Attia, Al‐Harthi, et al., 2021; Hassan et al., 2018). The physiological effects of fats and oils go beyond their energy‐providing function (Attia, Al‐Harthi, et al., 2021; Hassan et al., 2018). Fat‐soluble vitamins are contained in fats/oils. These ingredients have the potential to reduce pulverulence, enhance the absorption of fat‐soluble vitamins, improve diet palatability, boost immune response and disease resistance, and alter the synthesis of prostaglandin and thromboxane (Attia, Fulvia, et al., 2021). Studies have demonstrated that fat possesses an extra metabolic effect that may impact feed intake (FI), retention time of feed in the GIT, and consequently, nutrient digestion and absorption (Attia, Fulvia, et al., 2021; Calder, 2006; Cherian, 2011; Classen, 2017; Mateos et al., 1981, 1982). Early life energy requirements of broilers are partially satisfied by fats/oils (Geng et al., 2022).

The GIT uses mechanical and chemical processes to convert feed into constituent components, which are then transported by nutrient transporters to the intestinal epithelial cells. The present study investigated the impact of dietary energy density on the mRNA expression levels of PepT1, b^0,+^AT, B0AT, y^+^LAT1 and CAT1 in the jejunal portion. The PePT1 transporter has the capability to transport most dipeptides and tripeptides from the epithelial layer into intestinal cells, and the absorption process through this transporter is much more efficient and rapid than absorption via amino acid transporters (Wang et al., 2017). The b^0,+^AT transporter is the primary basic transporter located in the epithelial layer that transports lysine into intestinal cells and enables arginine to exit the cell and enter the intestinal lumen (Pineda et al., 2004). Furthermore, the transport of neutral and basic amino acids in the intestinal basolateral layer is facilitated by y^+^LAT1 (Honda et al., 2022). Neutral amino acids, particularly methionine, leucine, isoleucine and valine, are transported from the intestinal epithelial layer into intestinal cells primarily via B0AT (Honda et al., 2022). Lysine and arginine are transported in the intestinal basolateral layer by the CAT1 transporter, whose gene expression is regulated by factors, including nutrient type and density, hormones and growth factors (He et al., 2013; Pineda et al., 2004; Wang et al., 2017). Intestinal amino acid and peptide transporters are regulated by various factors, including genetic improvement, age, intestinal development, medicinal agents, form of feed, pathological states and nutrient density (Chen et al., 2005; Gilbert et al., 2008; He et al., 2013; Mahdavi et al., 2018; Morales et al., 2017; Osmanyan et al., 2018). Earlier studies suggest that the mRNA abundance of intestinal amino acid and peptide transporters in animals is regulated through various mechanisms, such as adaptive regulation theory (Hatzoglou et al., 2004), amino acid sensory pathways and amino acid‐responsive element (Fafournoux et al., 2000), peroxisome proliferator‐activated receptor‐α (PPARα; Shimakura et al., 2006), substrate supply to the intestine and intra‐enterocyte space (the 5´ upstream region of PepT1 contains substrate‐responsive elements; Chen et al., 2005; Mahdavi et al., 2018), increased mRNA stability and transcription rate (Adibi, 2003), plasma thyroxin (T4) levels and the state of intestinal villi (Mahdavi et al., 2018).

The density of dietary nutrients is a crucial factor in poultry production. Decreasing nutrient density can result in lower production costs, greater economic profit, and reduced environmental pollution (Zhang et al., 2021, 2020). Previous studies have investigated the effect of several factors, including gender and age of birds (Gilbert et al., 2007), protein and amino acid levels (Osmanyan et al., 2018), protein source (Gilbert et al., 2008, 2010) and physical form of feed (Mahdavi et al., 2018), on the mRNA expression of intestinal peptides and amino acid transporters in poultry. Most of the studies conducted to date have examined the impact of protein and amino acid levels on the expression of intestinal amino acid and peptide transporters in broilers and revealed their capacity to modify the expression of genes. However, the influence of fat level on the mRNA abundance of intestinal amino acid and peptide transporters has only been explored in one study conducted on pigs. Feng et al. (2014) examined the effects of four iso‐caloric and iso‐nitrogenous diets with different levels of fat and monosodium l‐glutamate (basal diet, high fat diet, basal diet with 3% monosodium l‐glutamate and high fat diet with 3% monosodium l‐glutamate) on growing pigs. The study demonstrated significant alterations in the expression of genes related to amino acid transporters (PepT1, EAAC1, LAT1, b^0,+^AT and ASCT2) in the jejunum, as well as changes in circulating and tissue amino acid pools upon dietary supplementation with fat or monosodium l‐glutamate.

To our knowledge, no previous research has evaluated the effect of dietary energy or fat level on the mRNA abundance of intestinal amino acid and peptide transporters in poultry. We hypothesized that there might be a relationship between the dietary energy level (variation in the dietary fat level) and the expression of intestinal peptide and amino acid transporters in broiler chickens. This study aimed to assess the impact of two levels of energy with constant levels of other nutrients on the performance, intestinal morphology and mRNA abundance of intestinal amino acid and peptide transporters of broiler chickens during the starter phase under normal rearing conditions.

## 2 MATERIALS AND METHODS

### 2.1 Experimental design

Two hundred and seventy one‐day‐old Ross 308 male broiler chickens were randomly allocated to two dietary treatments with 2950 and 2850 kcal metabolizable energy (ME)/kg of diet. Each treatment had 9 replicates of 15 chicks each. The experimental procedures were approved by the Ethical Animal Care and Use Committee of the Department of Animal Science of Razi University, Iran (IR.RAZI.REC.1400.066). Broilers were housed in 18 floor pens (1.0 × 1.0 m^2^) and reared according the Ross 308 broiler guidelines. Wood shavings were used as litter in this study. The diets and water were given ad libitum during the trial. In each pen, there were two nipple drinkers that provided the chicks with ad libitum access to water, whereas a single tray feeder was used to dispense food to the chicks. Corn–soybean meal‐based diet was formulated according to recommendations and offered in the form of crumble from 1 to 10 days of age. Hammer mill (GHM Hammer Mill, Zarin Persia Sanat Pars) with a 2‐mm screen was used to grind the cereal of the diet and mixed in a horizontal mixer (Paddle Mixer, Zarin Persia Sanat Pars). To prepare crumble feed, mash feed was processed at 75°C for 35 s in a conditioner and then pelleted with a die ring of 1.8 mm screen and 30‐mm thickness (Pellet Mill Progress 580, PTN). Within 10 min after pelleting, feed were cooled to a temperature of around 20°C. The moisture content of the pelleted feed was adjusted to the level of the mash diet. Whole pellets were crumbled with a length of 1760 mm and a diameter of 250 mm single roll pair crumbler (Model KRU7, Zarin Persia Sanat Pars). The room temperature was maintained at 33°C at the beginning of the experiment and then gradually reduced until reaching 30°C. The relative humidity of room was 50% ± 5%. The nutrient contents of the feedstuffs were analysed using NIRS DS2500 FOSS before the diets were formulated. Dry matter, crude protein, total ash and ether extract contents were determined according to (AOAC, 2005). The composition of the experimental diets is given in Table 1.

**TABLE 1:** Ingredients and calculated nutrients of the diets.

	ME (kcal/kg)	
Ingredients (g/kg)	2950	2850
Corn	518	518
Sunflower oil	29.0	17.2
Soybean meal	374	374
Corn gluten	30.0	30.0
dl‐Methionine	3.00	3.00
Lysine‐HCl	2.50	2.50
l‐Threonine	0.700	0.700
Dicalcium phosphate	22.8	22.8
Limestone	9.70	9.70
NaCl	2.20	2.20
Sodium bicarbonate	2.10	2.10
Vitamin premix^a^	2.50	2.50
Mineral premix^b^	2.50	2.50
Choline chloride	1.00	1.00
Bentonite	0.00	11.8
Calculated analysis		
Metabolizable energy (kcal/kg)	2950	2850
Crude protein (%)	23.2	23.2
Dig‐lysine (%)	1.26	1.26
Dig‐Met + Cys (%)	0.910	0.910
Dig‐Thr (%)	0.800	0.800
Calcium (%)	1.00	1.00
Available P (%)	0.480	0.480
Determined		
Dry matter (%)	91.3	90.8
Crude protein (%)	22.6	22.8
Ether extract (%)	5.73	4.28
Ash (%)	6.32	6.45

Abbreviation: ME, metabolizable energy.

^a^ Each kg of vitamin premix contains 6 mg retinol, 0.1 mg cholecalciferol, 72 mg tocotrienol, 16 mg menodione, 4 mg thiamine, 20 mg riboflavin, 6 mg pyridoxine, 0.08 mg cobalamins, 120 mg niacin, 50 mg Ca‐pantothenate, 2 mg folic acid and 0.08 mg biotin.

^b^ Each kg of mineral premix contains 140 mg of Cu, 179 mg of Zn, 12.5 mg of Mn, 0.5 mg of I, 0.25 mg of Co and 0.4 mg of Se.

### 2.2 Performance and jejunal morphometry

At 1 and 10 days of age, body weight was measured on a pen basis, whereas FI and feed conversion ratio (FCR) were calculated at the end of the starter phase. Energy efficiency ratio (EER) was calculated as grams of weight gain ×100/total Kcal ME intake. On day 10, one bird per replicate, which was closest to the mean body weight of pen, was selected and euthanized by cervical dislocation. A 3‐cm section of its jejunum, located 10 cm anterior to the Meckel's diverticulum, was excised. Tissue samples were fixed in 10% neutral buffered formalin, dehydrated in graded alcohol series, cleared in xylene, and embedded in paraffin. Samples were cut using a CUT4055 manual microtome (microTec Laborgeräte GmbH). Tissue sections were stained with hematoxylin–eosin and digitalized with a computer‐aided light microscope (Olympus BX51TF). Histological parameters, such as villus height (VH), from the tip to the villus‐crypt junction base; villus width (VW), at the half‐height of the villus; and crypt depth (CD), from the villus base to the crypt base, were recorded. These variables were measured from 10 well‐oriented villi and crypts. The villus height to crypt depth ratio (VCR) was also calculated. Furthermore, villus surface area (VSA) was calculated using the formula mentioned by Sakamoto et al. (2000):

$$\mathrm{VSA}=2\pi \times \left(\mathrm{VW}/2\right)\times \mathrm{VH}$$

where *𝛑* is 3.14, VW is the villus width, and VH is the villus height.

### 2.3 Real‐time PCR

The jejunum region has been established as the primary site for peptide and amino acid absorption in broiler chicks, as demonstrated by Chen et al. (2005) and Rodgers et al. (2012). On day 10, one bird per pen was randomly selected, and jejunal samples (5 cm anterior to the Meckel's diverticulum) were removed, rinsed with PBS, and placed in microtubes containing RNAlater solution for gene expression analysis purposes (Invitrogen). The intestinal samples were stored at −20°C until RNA extraction. The extraction of total RNA from a 200 mg jejunal sample was carried out in accordance with the manufacturer's recommended guidelines using Trizol Reagent (Invitrogen Corp.). One μg of RNA was treated and digested with DNase (Thermo Fisher Scientific) for 30 min at 37°C. The overall RNA quality was assessed by electrophoresis on agarose gel and then stained with ethidium bromide. Complementary DNA (cDNA) was synthesized from isolated RNA using the Easy cDNA Synthesis Kit (DENA Zist Asia). A negative control was prepared for all samples, which did not include the enzyme mix, to guarantee the absence of genomic contamination.

The polymerase chain reaction was conducted under the following conditions: an initial denaturation at 94°C for 2 min, followed by 40 cycles of 94°C for 15 s, 62°C for 15 s and 72°C for 20 s. All polymerase chain reactions were executed with the Amplitronyx Thermal Cycler (Nyx Technik, Inc.) using primers that were designed by Gilbert et al. (2008) and Zhang et al. (2017), with the specific primer sequences given in Table 2. β‐Actin was used as an internal control for target gene expression normalization. All samples were subjected to real‐time PCR in triplicate, with each reaction comprising 1 μL of diluted cDNA samples (1:5), 2 μL SYBR Green Master Mix (DENA Zist Asia), 5 μL H_2_O and 1 μL of both forward and reverse primers, resulting in a total reaction volume of 10 μL. The LightCycler96 RT‐PCR System (Roche) was utilized to carry out the protocol outlined as follows: (1) one cycle at 95°C for 180 s as preincubation program; (2) 40 repeated cycles (15 s at 95°C, 15 s at 62°C and 15 s at 72°C) for amplification; and (3) one cycle at 95°C for 10 s, at 65°C for 1 min and at 97°C for 1 s as melting program. The relative mRNA expression change of the target gene relative to β‐actin was calculated using the following formula (Zeng et al., 2011):

$$R=2^{\left[C_{T(\beta -\mathrm{actin})}-C_{T(\mathrm{test})}\right]}$$

where *R* is the relative expression ratio, and *C*_T_ is the threshold cycle.

**TABLE 2:** Sequences of real‐time PCR primers used for selected genes.

SLC name	Gene	GenBank ID	Primer sequence sense/antisense	Amplicon size (bp)
SLC15A1	PepT1	NM_204365.1	CCCCTGAGGAGGATCACTGTTGGCAGTT/CAAAAGAGCAGCAGCAACGA	66
SLC7A9	b^o,+^AT	NM_001199133.1	CAGTAGTGAATTCTCTGAGTGTGAAGCT/GCAATGATTGCCACAACTACCA	99
SLC7A1	CAT1	NM_001145490.1	CAAGAGGAAAACTCCAGTAATTGCA/AAGTCGAAGAGGAAGGCCATAA	75
SLC6A19	B^o^AT	XM_419056.4	GGGTTTTGTGTTGGCTTAGGAA/TCCATGGCTCTGGCAGAGAT	112
SLC7A7	y^+^LAT1	XM_418326.4	CAGAAAACCTCAGAGCTCCCTTT/TGAGTACAGAGCCAGCGCAAT	110
	β‐Actin	NM_205518.1	GTCCACCGCAAATGCTTCTAA/TGCGCATTTATGGGTTTTGTT	110

Abbreviation: SLC, solute carrier family.

### 2.4 Statistical analysis

The pen was the experimental unit for the growth performance data, whereas the bird was the experimental unit for the analysis of intestinal morphology and gene expression of peptide and amino acid transporters. The data were analysed in a completely randomized design with the general linear procedure of SAS software Version 9.2 (SAS Institute, Cary, NC, 2017) using the following statistical model:

$$Y_{ij}=\mu +T_{i}+e_{ij}$$

where *Y_ij_* is the dependent variable, *μ* is the overall mean, *T_i_* is the effect of dietary energy levels and *e_ij_* is the random error. When significant difference was detected, means were separated using Duncan's multiple range test. Statistical significance level was set at *p* ≤ 0.05.

## 3 RESULTS

### 3.1 Growth performance

The results of this experiment indicated that a reduction of 100 kcal ME/kg in dietary energy did not impair the growth performance of broilers in the starter phase (*p* > 0.05, Table 3). Furthermore, the reduction in dietary ME had no impact on the EER.

**TABLE 3:** Effects of different levels of metabolizable energy of starter diet on the growth performance of broilers.

ME (kcal/kg)	Body weight gain (g)	Feed intake (g)	FCR	Energy efficiency ratio
2950	191.1 ± 19.1	217.4 ± 11.8	1.15 ± 0.201	29.8 ± 3.24
2850	183.0 ± 19.1	225.0 ± 10.3	1.23 ± 0.138	28.6 ± 2.40
SEM	5.82	4.16	0.0481	0.582
*p*‐Value	0.312	0.193	0.186	0304

*Note*: Results are shown as mean  ±  SD. *N*  =  9 per group.

Abbreviations: FCR, feed conversion ratio; ME, metabolizable energy; SEM, standard error of the mean.

### 3.2 Intestinal amino acid and peptide transporters

This study demonstrated a significant association between dietary energy level and the mRNA abundance of some intestinal amino acid transporters (Table 4), with a decrease in dietary energy level resulting in a significant reduction in the mRNA expression of b^0,+^AT and B0AT transporters (*p* ≤ 0.05), whereas the mRNA levels of PepT1, y^+^LAT1 and CAT1 remained unaffected (*p* > 0.05).

**TABLE 4:** Effects of different levels of metabolizable energy of starter diet on mRNA abundance (×10^−3^) of peptide and amino acid transporters of broilers.

ME (kcal/kg)	PepT1	b^0,+^AT	CAT1	B^0^AT	y^+^LAT1
2950	0.781 ± 0.199	141.0 ± 48.9^a^	1.16 ± 0.323	124.7 ± 25.3^a^	2.08 ± 0.861
2850	0.826 ± 0.145	109.4 ± 21.0^b^	0.828 ± 0.258	85.4 ± 22.4^b^	1.84 ± 0.606
SEM	0.0281	7.70	0.0623	5.82	0.178
*p*‐Value	0.376	0.0437	0.541	0.0002	0.451

*Note*: Within a column, means with different superscripts differ significantly (*p* < 0.05). Results are shown as mean  ±  SD. *N*  =  9 per group. Nine intestinal samples per treatment and two measurements per sample were measured.

Abbreviations: ME, metabolizable energy; PepT1, peptide; SEM, standard error of the mean.

### 3.3 Intestinal morphology

Based on the data in Table 5, it can be concluded that dietary energy level did not significantly alter the intestinal morphological indices, such as VH, VW, CD, VCR and VSA (*p* > 0.05).

**TABLE 5:** Effects of different levels of metabolizable energy of starter diet on the intestinal histomorphology of broilers.^a^

ME (kcal/kg)	Villus height (μm)	Villus width (μm)	Crypt depth (μm)	VCR	VSA (×10^−3^ μm^2^)
2950	535.0 ± 38.2	79.4 ± 4.48	203.7 ± 17.5	2.63 ± 0.181	133.4 ± 11.5
2850	494.9 ± 35.4	85.1 ± 7.89	187.0 ± 14.4	2.65 ± 0.167	132.5 ± 17.5
SEM	12.0	1.99	5.13	0.0534	4.14
*p*‐Value	0.176	0.461	0.235	0.683	0.426

*Note*: Results are shown as mean  ±  SD. *N*  =  9 per group and 10 villi and crypts per sample.

Abbreviations: ME, metabolizable energy; SEM, standard error of the mean; VCR, villus height to crypt depth ratio; VSA, villus surface area.

^a^ Within a column, means with different superscripts differ significantly (p < 0.05).

## 4 DISCUSSION

Carbohydrates and oils/fats serve as the main energy sources for poultry. Among these, oils/fats are more advantageous due to their low heat increment, greater calorific effect and inclusion of fat‐soluble vitamins, resulting in better nutrient utilization (Gao et al., 2021). Despite their advantages, they are typically costly. Additionally, in many developing countries like Iran, fats/oils are imported to fulfil the needs of both human and animal consumption. It is possible to decrease the dietary energy level without adversely affecting growth performance. Hence, in this study, we lowered the energy level of the diet by reducing its fat content. The findings indicated that reducing the dietary energy density did not have any adverse effects on the FI, body weight gain (BWG), FCR and EER of broiler chickens. Similarly, it has been shown that a reduction in feed energy level by 75 kcal ME/kg diet (from 3000 to 2925 kcal/kg) did not affect the FI, BW and FCR of broiler chickens during the starter period (Wan et al., 2020). In addition, Vieira et al. (2006) found that adjusting the dietary energy level (from 2870 to 3100 kcal ME/kg) did not result in any significant change in the BWG of broiler chicks at 7 days of age. However, Hu et al. (2018) found that the BWG of broiler chickens fed a diet with 3050 kcal ME/kg was lower compared with those fed a diet containing 3200 kcal ME/kg. It was demonstrated by Ghahremani et al. (2016) that reducing the dietary ME by 100 kcal/kg did not have any significant effects on the BWG, FI and FCR of Cobb broiler chickens, but a further decrease resulted in negative consequences. The results pertaining to EER were consistent with the study conducted by Kamran et al. (2008), who found that manipulating dietary energy and protein levels during the starter period did not affect the EER of broiler chickens at 10 days of age.

The findings of the current study also revealed that changing the dietary energy density did not alter the jejunal morphological indices, including VH, VW, CD, VCR and VSA. Intestinal development serves as an indicator of animal health and has a significant impact on the efficiency of nutrients digestion and absorption (Aruwa et al., 2021). The production of new epithelial cells occurs within the crypt, and these cells migrate and differentiate as they travel along the villi. According to Ghahremani et al. (2016), a decrease of 100 kcal ME/kg in dietary energy did not affect the VH, CD or VCR in the duodenum of broilers. However, further reduction in dietary energy (180 kcal ME/kg diet) had a harmful effect on the intestinal morphology. Kim et al. (2019) conducted a study wherein Pekin ducks were fed diets containing 2950, 3000, 3050, 3100 and 3150 kcal ME/kg from 21 to 42 days of age. The results of the study revealed that the dietary energy density had no effect on the jejunal VH and CD at 28, 35 and 42 days of age. In contrast, Zanganeh et al. (2015) found that increasing dietary energy by 100 kcal/kg led to an increase in the VH and VCR, as well as deeper ileal crypts, whereas the CD of the jejunum villi and the VH and VCR of the ileum villi remained unaffected. Differences in the experimental conditions, such as the length of the experiments, dietary energy level, age, gender and strain of broiler chickens, could account for the variation between the results of our study and previous research.

In this study, the quantity of dietary oil was modified to alter the dietary energy level. We observed that birds fed a low‐energy diet (with the same nutrient contents as the control diet) consumed approximately 10% more feed. Consequently, it was assumed that the substrate levels, particularly amino acids and peptides, in the gut were higher in birds fed the low‐energy diet. The situation is similar to that in which the nutrient concentrations of the diet are increased, which subsequently leads to a higher availability of substrates for absorption in the intestine. The present study found that the mRNA expression of b^0,+^AT and B0AT transporters decreased with a reduction in dietary energy level, but there were no changes in the mRNA levels of PepT1, y^+^LAT1 and CAT1. The findings of this study are in agreement with those of Feng et al. (2014), who observed that a high‐fat diet increased the mRNA expression of b^0,+^AT in the jejunum of pigs, but the mRNA level of PepT1 was not affected by the dietary fat level. Novak et al. (2012) observed that dietary fatty acids can conserve amino acids for peptide and protein synthesis, whereas Hiroaki (2006) reported that sulphur‐containing amino acids can modulate lipid metabolism. The intestinal microbiota has been shown to exert a significant influence on the synthesis and degradation of nutrients, including amino acids (Davila et al., 2013). Moreover, there is evidence to suggest that dietary fat can alter the composition of the gut microbiota (Turnbaugh 2012; Moreira et al., 2012; Feng et al., 2014). Thus, changes in the gut microbiota could conceivably contribute to the up‐ or down‐regulation of peptide and amino acid transporters in the jejunum by affecting the synthesis and degradation of amino acids or by other mechanisms that are yet to be uncovered. Given its role as a brush border transporter, a decrease in the expression of b^0,+^AT would likely result in a reduction in uptake and depletion of neutral and basic amino acids from the cellular stores. In contrast, as B0AT is a basolateral transporter, a decrease in its expression would decrease the efflux of neutral amino acids from the cell and consequently increase cellular reserves. The co‐occurrence of reduced expression of b^0,+^AT and increased intestinal substrate levels observed in this study can be explained by the adaptive regulation theory. This theory postulates that amino acid transporter activity is stimulated by substrate scarcity and inhibited by excess substrate (Hatzoglou et al., 2004; Majumder et al., 2009). Regarding B0AT, it is probable that the intestinal concentration of neutral and basic amino acids increased to such an extent that it compensated for the decrease in b^0,+^AT expression, leading to an elevation in the intra‐enterocyte concentration of these amino acids. Consequently, the expression of B0AT decreased in accordance with adaptive regulation theory. As mentioned previously, dietary fats/oils have the extra metabolic effect. Studies have demonstrated that dietary fat can prolong the feed retention time in the GIT, potentially enhancing the digestion and absorption of nutrients (Mateos et al., 1981, 1982). The extra metabolic effect of fats and oils is a plausible explanation for the decreased expression of genes b^0,+^AT and B0AT; however, the exact metabolic pathways involved are currently unidentified and necessitate additional research. There are several factors that can influence the expression of transporter genes, and further research is needed to investigate their effects. For instance, fatty acids can activate the PPARα, which is a nuclear receptor (Shimakura et al., 2006).

## 5 CONCLUSION

The findings of the present study suggest that a reduction in the energy density of the starter diet by 100 kcal ME/kg via a decrease in its oil content can alter the mRNA expression of intestinal amino acid transporters in broiler chickens during the first 10 days of age without affecting their growth performance, EER or intestinal morphometric structure. Further research is needed to investigate the impact of a broader range of energy levels and the interaction between dietary energy and amino acid levels on the profitability, expression of various nutrient transporters, intestinal morphology, nutrient digestibility and gut microbiota, which could provide more insights into the optimal nutrition and feeding strategies for broiler chickens.

## AUTHOR CONTRIBUTIONS

All authors contributed to the study conception and design. Material preparation, data collection and analysis were performed by Reza Mahdavi, Shahab Ghazi Harsini and Ali Hossein Piray. The first draft of the manuscript was written by Reza Mahdavi and all authors commented on previous versions of the manuscript. All authors read and approved the final manuscript.

## CONFLICT OF INTEREST STATEMENT

The authors declare no conflicts of interest.

## FUNDING INFORMATION

None.

## ETHICS STATEMENT

This study was approved by the Animal Care and Use Committee of Razi University, with the approval number (IR.RAZI.REC.1400.066).

### PEER REVIEW

The peer review history for this article is available at https://www.webofscience.com/api/gateway/wos/peer‐review/10.1002/vms3.1364.

## Data Availability

The data that support the findings of this study are available from the corresponding author upon reasonable request.
